# Supplementary material for: Time-Limited Therapy with Belatacept in Kidney Transplant Recipients
Source: J Clin Med. 2022 Jun 6;11(11):3229. doi: 10.3390/jcm11113229 (PMC9181670; doi:10.3390/jcm11113229)
Supplement: Supplementary file 1 [file jcm-11-03229-s001.zip › jcm-1695435-supplementary.pdf]

Supplemental Figure S1. Tacrolimus trough level after belatacept discontinuation (n = 25) Each dot representing mean tacrolimus trough level at corresponding time point, and error bar standard deviation.

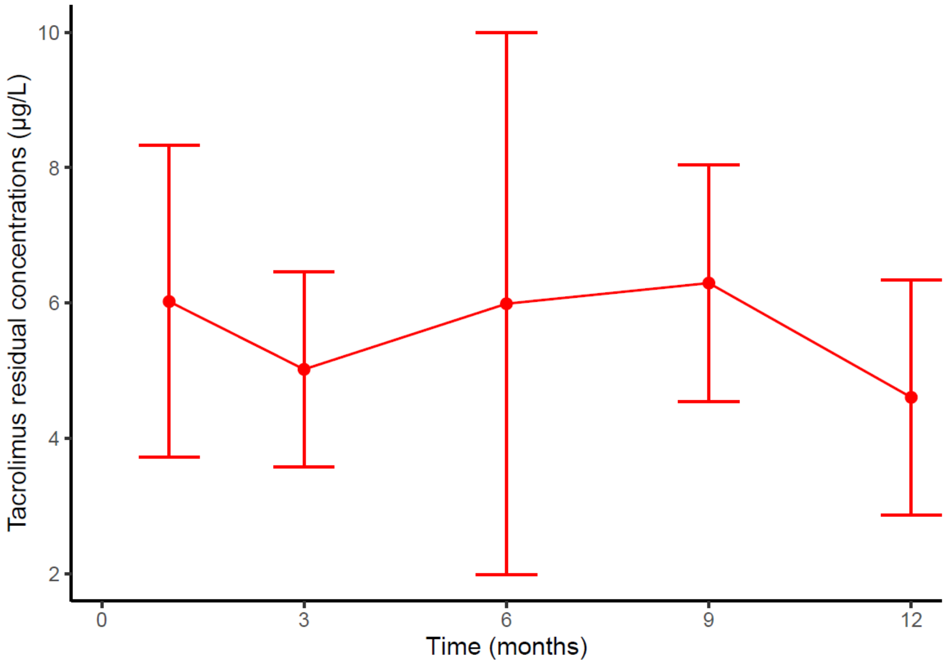

Supplemental Table S1. Gene Ontology enrichment analysis (biological process) of Differential Expressed Genes between Day 0 and 3-months post belatacept withdrawal.

Gene ontology (biological process) enrichment analysis of the 29 up-regulated DEGs

Index	Gene Ontology Term	P-value	Adjusted p-value	Odds Ratio	Combined score
1	regulation of respiratory burst involved in inflammatory response (GO:0060264)	0.005986	0.04995	217.09	1111.12
2	regulation of lyase activity (GO:0051339)	0.005986	0.04995	217.09	1111.12
3	negative regulation of T cell receptor signaling pathway (GO:0050860)	0.0002331	0.005657	106.73	892.73
4	mitochondrial electron transport, NADH to ubiquinone (GO:0006120)	0.00001349	0.001200	79.13	887.32
5	macromolecule biosynthetic process (GO:0009059)	0.007179	0.05726	173.66	857.29

Gene ontology (biological process) enrichment analysis of the 40 down-regulated DEGs

Index	Gene Ontology Term	P-value	Adjusted p-value	Odds Ratio	Combined score
1	negative regulation of leukocyte degranulation (GO:0043301)	0.0001535	0.007843	142.51	1251.43
2	SRP-dependent cotranslational protein targeting to membrane (GO:0006614)	3.229e-8	0.00001286	39.43	680.15
3	cotranslational protein targeting to membrane (GO:0006613)	4.197e-8	0.00001286	37.63	639.24
4	protein targeting to ER (GO:0045047)	7.268e-8	0.00001485	34.13	560.93
5	positive regulation of amine transport (GO:0051954)	0.01046	0.1002	121.67	554.88
